# Clinical and immunological biomarkers in hypereosinophilic syndrome: the second step after diagnostic algorithms

**DOI:** 10.3389/fmed.2025.1600728

**Published:** 2025-07-02

**Authors:** David Longhino, Ilaria Baglivo, Maria Antonietta Zavarella, Stefania Colantuono, Chiara Laface, Gabriele Lucca, Laura Bruno, Fabio Romano Selvi, Vincenzo Patella, Aikaterini Detoraki, Rosa Buonagura, Caterina Tatarelli, Barbara Moscatelli, Serena D’Avelli, Elisabetta Abruzzese, Elisabetta Greco, Antonio Gasbarrini, Livio Pagano, Marianna Criscuolo, Sabrina Giammarco, Cristiano Caruso

**Affiliations:** ^1^UOSD Allergology and Clinical Immunology Unit, Fondazione Policlinico Universitario ‘A. Gemelli’ IRCCS, Università Cattolica del Sacro Cuore, Rome, Italy; ^2^Centre for Digestive Diseases (CEMAD) and Gastroenterology Unit, Fondazione Policlinico Universitario A. Gemelli, IRCCS, Rome, Italy; ^3^UOSD DH Internal Medicine and Digestive Disease, Fondazione Policlinico A. Gemelli, IRCCS, Rome, Italy; ^4^Division of Allergy and Clinical Immunology, Department of Medicine, “Santa Maria della Speranza” Hospital, Salerno, Italy; ^5^Division of Internal Medicine and Clinical Immunology, Department of Internal Medicine and Clinical Complexity University of Naples Federico II, Naples, Italy; ^6^Department of Hematology, S. Andrea Hospital, Rome, Italy; ^7^Department of Internal Medicine, Gemelli Isola, Rome, Italy; ^8^ASL Frosinone, Pneumology Unit, Frosinone, Italy; ^9^Hematology, Sant’Eugenio Hospital, Tor Vergata University, Rome, Italy; ^10^UOC Reumatologia, Dipartimento di Medicina dei Sistemi, Università di Roma “Tor Vergata”, Rome, Italy; ^11^Department of Hematology, Fondazione Policlinico Universitario A. Gemelli, IRCCS, Università Cattolica del Sacro Cuore, Rome, Italy

**Keywords:** eosinophils - immunology, free light chain (FLC), HES, biomarkers, ECP

## Abstract

**Background:**

Idiopathic hypereosinophilic syndrome currently represents a major unmet need for all medical specialties dealing with this disease. Markers capable of characterising the wide variability of its clinical presentation are currently lacking.

**Objective:**

This study aims to evaluate a panel of possible markers in idiopathic hypereosinophilic syndrome.

**Methods:**

In this pilot prospective single-centre cohort study, we analysed clinical (age, years of disease, steroid therapy) and laboratory (absolute eosinophil count, total IgE antibodies, IgE antibodies against Staphylococcus aureus enterotoxins, serum eosinophil cationic protein, serum immunoglobulin free light chains k and λ and their ratio) data obtained from 21 patients suffering from idiopathic hypereosinophilic syndrome from June 2023 to December 2024.

**Results:**

Mean absolute eosinophilic count was 3758.57 cells/μL. 17 patients were receiving treatment with > 7.5 mg of prednisone or equivalent at the time of the diagnosis. 13 patients had positive Staphylococcus aureus enterotoxins IgE, while the mean total serum IgE was 241.64 kU/L. We observed a high serum eosinophil cationic protein value as well as a high serum κ free light chain, while serum λ and κ/λ were normal. Patients with higher absolute eosinophilic count had higher eosinophil cationic protein levels (*p* < 0.05), such as higher steroid consumption (*p* < 0.05). In addition, we found a strong association between high κ free light chain levels and high previous steroid use and with Staphylococcus aureus enterotoxins IgE positivity.

**Conclusion:**

Our results could increase the number of possible biomarkers for risk stratification in idiopathic hypereosinophilic syndrome.

## Introduction

Hypereosinophilia (HE) is defined when ≥ 1.5 eosinophils × 10^9^/L on two examinations in peripheral blood with an interval ≥ 2 weeks occur; therefore, when HE is associated with organ damage and/or disfunction attributable to HE and other conditions responsible for that organ damage and/or disfunction are ruled out, a diagnosis of hypereosinophilic syndrome (HES) is established. Idiopathic hypereosinophilic syndrome (I-HES) occurs when other causes of HES cannot be found (1).

The North American Surveillance, Epidemiology and End Results cancer registry and the International Classification of Diseases for Oncology, estimated the average annual incidence of HES between 2001 and 2005 to be 0.36 new cases per million inhabitants per year. However, this study did not include details of the different subtypes of HES (2).

In a recent study conducted in the United Kingdom, Requena et al. (3) analyzed primary care data from the Clinical Practice Research Datalink between 2010 and 2018; during this period HES incidence ranged from 0.04 to 0.17 per 100,000 person/years, while prevalence ranging from 0.15 to 0.89 cases per 100,000 persons (4). Incidence and prevalence of I-HES therefore remain unknown to date.

In the era of precision medicine, I-HES must become not only a diagnosis of exclusion of the most common reactive and clonal causes of hypereosinophilia, but a full-fledged clinical entity with its own diagnostic algorithm, biomarkers, clinical scores and targeted therapies. Currently, despite being a relatively recent clinical entity, HES already has diagnostic algorithms (1, 5) and effective therapies (6).

Even nowadays, clinical scores and biomarkers capable of defining disease progression for this condition instead remain unmet needs and represent the next step in the management of this disease.

Indeed, distinction of silent disease from active disease, detection of irreversible organ damage and tools capable of stratifying disease severity are currently lacking. Moreover, patient-reported outcomes do not exist. It is therefore crucial to identify potential biomarkers capable of predicting or monitoring organ damage over time. Several biomarkers have been proposed for monitoring disease activity (7), but they are still not validated or have not entered clinical practice.

Traditional biomarkers such as serum interleukin-5 (IL-5), thymus and activation-regulated chemokine (TARC/CCL17), total IgE, vitamin B12, and tryptase levels have been evaluated for their prognostic significance. Elevated IL-5 and TARC levels suggest a lymphocytic variant of HES (L-HES), characterized by aberrant T-cell clones producing eosinophilopoietic cytokines. However, these markers lack specificity and are not universally applicable across all I-HES subtypes (8–10).

Recent research has highlighted the potential of eosinophil granule proteins—major basic protein (MBP), eosinophil cationic protein (ECP), eosinophil peroxidase (EPO), and eosinophil-derived neurotoxin (EDN)—as indicators of disease activity. These proteins, released during eosinophil activation, correlate with tissue damage and may serve as more direct measures of disease burden (7).

A nationwide Japanese survey identified several clinical factors associated with poorer outcomes in I-HES patients: age over 50 years, hemoglobin levels below 12 g/dL, activated partial thromboplastin time exceeding 34 s, presence of dyspnea, thrombotic tendencies, and renal failure. Additionally, renal failure, splenomegaly, and pulmonary abnormalities were linked to shorter durations of response to corticosteroid therapy (10).

Cardiac involvement remains a critical determinant of prognosis, with early cardiac disease and thromboembolic events contributing to increased mortality. High peak eosinophil counts, and rapid disease progression are also associated with adverse outcomes (8, 11).

In the management of I-HES, there remains a critical lack of validated clinical and immunological biomarkers that can reliably assess disease activity, predict therapeutic response, or stratify patients based on disease severity.

The aim of this study is to explore the clinical utility of selected immunological biomarkers—including ECP, free light chains (FLCs) κ and λ, and Staphylococcus aureus enterotoxin (SEs)-specific IgE—in a real-world cohort of patients with I-HES. Specifically, we seek to investigate potential associations between these biomarkers and clinical variables such as systemic corticosteroid use and disease duration, in order to generate hypothesis-driven evidence for their future integration into disease stratification models and outcome prediction tools.

Herein, we report our comprehensive clinical experience reporting possible new clinical biomarkers analysed in 21 patients with I-HES.

## Materials and methods

In this pilot prospective single-center cohort study, we evaluated clinical and laboratory data of 21 patients suffering from I-HES referred to our Allergy and Clinical Immunology Unit of Policlinico Universitario A. Gemelli IRCCS of Rome, Italy, from June 2023 to December 2024.

Inclusion criteria were age ≥ 18 years and a prevoius I-HES diagnosis, meanwhile exclusion criteria were all other possible aetiologies of HES (secondary, clonal or familial) and inability to carry out laboratory analyses or to continue the follow-up.

As primary outcome, after ruling out reactive and hematological causes of hypereosinophilia and diagnosing I-HES in these patients, we evaluated possible biomarkers of organ damage and disease course; in particular, we analysed clinical (age, years of disease, steroid therapy) and laboratory (absolute eosinophil count (AEC), total IgE antibodies, IgE antibodies against Staphylococcus aureus enterotoxins (SEs), serum eosinophil cationic protein (ECP), serum immunoglobulin free light chains (FLCs) k and λ and their ratio) data.

SEs sensitization was detected by ImmunoCAP (ThermoFisher Scientific/Phadia, Uppsala, Sweden).

To analyze FLCs, the gathered samples underwent centrifugation at 2500 g for 10 min, and the serum was separated into aliquots before being frozen at −80°C for storage until analysis. The samples were thawed a single time, allowed to reach room temperature, and analyzed immediately. The analysis was conducted by an operator who was unaware of the patient’s clinical history. Each sample was processed using the OPTILITE (The Binding Site, Birmingham, United Kingdom) analyzers, following the manufacturer’s guidelines. Reference ranges for κ FLCs are 3.3–19.4 mg/L and 5.7–26.3 mg/L for λ FLCs respectively (12). A ratio of *κ/λ* < 0.26 or > 1.65 is considered abnormal, according to the manufacturer’s recommendations.

The sample was described in its clinical and demographic characteristics through descriptive statistics techniques. Qualitative variables have been presented as absolute frequencies and percentages, while quantitative variables have been summarized with mean and standard deviations. The normality of data has been verified with the Kolmogorov–Smirnov test. Proportions were compared applying Fisher’s exact test. Given the small sample size of our cohort (*n* = 21), no *a priori* sample size calculation was performed. A *p-value* < 0.05 was considered statistically significant. All the statistical analyses have been performed with Statistical Package for the Social Sciences (SPSS) statistical software (Released 2017, IBM SPSS Statistics for Windows, Version 25.0; IBM Corp., Armonk, NY).

## Results

21 patients (14 males and 7 females, mean age of 53.62 years) were evaluated. The mean AEC was 3758.57 cells/μL. 17 patients were receiving treatment with > 7.5 mg of prednisone or equivalent at the time of the diagnosis. The mean years of disease were 5.86. 13 patients had positive SEs, while the mean total serum IgE was 241.64 kU/L. We observed a high serum ECP value (mean 92.1 μg/L) as well as a high serum κ FLCs (mean 23.72 mg/L), while serum λ FLCs and κ/λ were normal (18.34 mg/L and 1.28, respectively). Patients with higher AEC had higher ECP levels (*p* < 0.05), such as higher steroid consumption (*p* < 0.05). In addition, we found a strong association, although not statistically significant, between high κ FLCs levels and high previous steroid use, and between high κ FLCs levels and SEs positivity. Clinical and laboratory data are reported in Table 1.

**TABLE 1:** Clinical and laboratory data of the patients.

	Age (years)	Length of disease (years)	Prednisone (< / > 7.5 mg)	
	Mean (SD)		*n (%)*	
**Clinical data**				
	53.62 (21.68)	5.86 (4.66)	> 7.5 mg: 4 (19%)	
			< 7.5 mg: 17 (81%)	
	**Baseline eosinophils (cells/μL)**	**SEs IgE (positive/negative)**	**Total IgE (kU/L)**	
	**Mean (SD)**	***n (%)***	**Mean (SD)**	
**Laboratory data**				
	3758.57 (2538.65)	Positive: 13 (61.9%) Negative: 8 (38.1%)	241.64 (455.55)	
	ECP (μg/L)	FLC k (mg/L)	FLC λ (mg/L)	Ratio k/λ
	Mean (SD)	Mean (SD)	Mean (SD)	Mean (SD)
	92.1 (76.21)	23.72 (7.85)	18.34 (5.37)	1.28 (0.21)

ECP, eosinophilic cationic protein; FLC, free light chain; SEs, *Staphylococcus aureus* enterotoxins.

## Discussion

Hypereosinophilic syndrome, and in particular I-HES, still needs a considerable amount of in-depth research nowadays, covering all facets of the disease. The aim of this study is to enrich the meagre and not yet exhaustive list of possible biomarkers of damage in I-HES. In particular, we evaluated both possible biomarkers of systemic and organ damage; in fact, the immunological strength characterized by hypereosinophilia could potentially involve all organs. Biomarkers of systemic disease include AEC, serum IL-5, and ECP, all related to eosinophils activity and their triggered damage (7); biomarkers of organ damage, instead, potentially include any laboratory and radiology test altered by this disease.

The average age of the patients analysed was 53.62 years, with an average time between onset of symptoms and diagnosis of 5.86 years. These data are in line with those already described in literature (9). Interestingly, 80.95% of patients at the time of diagnosis received > 7.5 mg of prednisone or equivalent.

Eosinophil-related biomarkers (such as AEC, ECP, serum IL-5) have a significant role in this condition; in particular, AEC could positively correlate with disease activity and response to anti IL-5 therapies, while soluble mediators seem to correlate with active disease (7). In our patients, patients with elevated AEC values positively correlated with higher ECP values (*p* < 0.05), as well with a higher steroid consumption (*p* < 0.05). In a recent article, elevated serum ECP levels have been associated with poor disease control in chronic spontaneous urticaria (CSU), suggesting its potential as a predictive biomarker; in our study, we found a markedly high levels of ECP in patients suffering from I-HES, highlighting the varying roles of ECP across different eosinophil-associated disorders (13). IL-5 is the most important cytokine for the production, regulation and survival of eosinophils; this cytokine is blocked by mepolizumab, a monoclonal antibody directed against IL-5, which is the only approved biological therapy for I-HES so far (6).

Our attention was particularly focused on biomarkers not yet described in literature in association with I-HES.

While traditionally associated with plasma cell disorders and autoimmune diseases, recent literature suggests FLCs may also reflect epithelial immune activation. Elevated κ FLCs have been noted in asthma, allergic rhinitis, and eosinophilic esophagitis. Our study extends these findings to I-HES, where κ FLCs were elevated in the majority and trended with disease severity markers. SEs-specific IgE, linked to chronic airway inflammation and epithelial barrier dysfunction, was positive in over 60% of patients and showed potential association with κ FLCs, suggesting overlapping mechanisms of immune activation.

Immunoglobulins play a crucial role in host defence mechanisms. In healthy individuals, plasma cells produce five classes of immunoglobulins. They are composed of two identical heavy chains and two identical light chains, which are connected by disulfide bonds to form tetrameric structures. The class and the subclass of immunoglobulins, as well as their biological functions, are determined by the C-terminal region of the heavy chain. The N-terminal regions of both heavy and light chains contain hypervariable regions that are essential for antigen binding. Some immunoglobulin light chains are not incorporated into tetrameric structures and are instead secreted as free light chains (FLCs) (14). Elevated serum levels of FLCs are observed not only in hematological diseases in which a clonal expansion of plasma cells is observed, but also in autoimmune diseases such as systemic lupus erythematosus, rheumatoid arthritis, systemic sclerosis, and Sjögren syndrome; moreover, changes in their levels are associated with disease activity (15–20).

It was observed that FLCs are involved in hypersensitivity reactions (21), and concentration of kappa FLCs is elevated in asthmatic patients (22). Apart from asthma, FLCs are increased in allergic and non-allergic rhinitis nasal mucosa (23).

Furthermore, an increased FLCs value was observed in female patients with eosinophilic oesophagitis when compared to male patients with the same condition (24).

Interestingly, we found a strong association, although not statistically significant, between high κ FLCs levels and high previous steroid use, and between high κ FLCs levels and SEs positivity, suggesting a potential link between epithelial barrier dysfunction, immune activation, and systemic disease burden in I-HES. These findings raise the hypothesis that κ FLCs could serve as surrogate markers of inflammatory activity in patients with a more severe or treatment-refractory phenotype, warranting further investigation in larger, prospective cohorts.

SEs positivity has been associated with the severity of certain diseases such as atopic dermatitis, chronic rhinosinusitis with nasal polyps, and asthma (25). Being present both in skin and in respiratory disease, SEs positivity might suggest its role in pathologies where an epithelial barrier damage is present, but further studies are needed.

In order to develop a patient-based diagnostic and therapeutic algorithm, biomarkers capable of framing the patient’s disease state and possibly predicting disease progression will have to be researched and these should enter clinical practice. However, being a recently defined entity, further studies are needed, as well as, to underline the growing concern about this disease, a disease activity score and patient- and clinician- reported outcome are expected to enter clinical practice (26).

## Conclusion

Our results could increase the number of possible biomarkers for risk stratification in I-HES, in addition to those already described in the Literature, helping to better characterize patients suffering from this disease for a more appropriate individualized clinical management.

## Data Availability

The original contributions presented in the study are included in the article/supplementary material, further inquiries can be directed to the corresponding author.
